# A retrospective case series of electroconvulsive therapy in the management of depression and suicidal symptoms in adolescents

**DOI:** 10.1002/brb3.2795

**Published:** 2022-10-19

**Authors:** Xiaolu Chen, Yixiao Fu, Qianhong Zou, Yiting Zhang, Xiaoyue Qin, Yu Tian, Yu Yan, Qibin Chen, Lei Zou, Bangshu Zhao, Xiao Li

**Affiliations:** ^1^ The First Branch The First Affiliated Hospital of Chongqing Medical University Chongqing China; ^2^ Department of Psychiatry The First Affiliated Hospital of Chongqing Medical University Chongqing China; ^3^ Department of the First Clinical Medicine Chongqing Medical University Chongqing China; ^4^ Information Center The First Affiliated Hospital of Chongqing Medical University Chongqing China; ^5^ Department of Anesthesiology The First Affiliated Hospital of Chongqing Medical University Chongqing China

**Keywords:** adolescents, depression, efficacy, electroconvulsive therapy (ECT), side effect, suicide

## Abstract

**Objective:**

Major depressive disorder (MDD) with suicidal symptoms is common in adolescents. Electroconvulsive therapy (ECT) is highly effective in the treatment of MDD. We have described its use and outcome in a case series of adolescents with depression and suicidal symptoms receiving ECT.

**Methods:**

We analyzed 362 adolescents aged from 12 to 17 who had received ECT between year 2015 and 2021. A total of 278 subjects were found to meet the inclusion criteria, where depressive symptoms were assessed by HDRS and suicidal symptoms were assessed by HDRS item 3. Their sociodemographic, clinical, and treatment information were retrieved through these records for this study.

**Results:**

The mean ± SD age of subjects was 15.41 ± 1.50 years and male sex was 14.7% (*n* = 41). Comorbid diagnoses were present in 104 patients (37.4%). The ECT sessions ranged from 6 to 12 times. All the patients took antidepressants, with sertraline (*n* = 182; 65.5%) being the most widely used. Majority of patients also received benzodiazepines. ECT was significantly effective in adolescents with depression and suicidal symptoms in evaluation by HDRS, HDRS item 3, CGI‐S (*p* < .001) pre/post‐ECT. The response rate of MDD patients was 52%, with suicidal ideation (SI) at 49%, and 54% in MDD with suicide attempt (SA). The change of CGI‐S scores showed no significant differences between various subgroups of sex and comorbid (*p*>.05), but there were significant differences between subgroups of suicidal symptoms (*p* < .001). ECT was generally safe with subjective memory complaint (*n* = 189, 68.0%), headache (*n* = 150, 54.0%), body pain (*n* = 28, 10.1%), delirium (*n* = 95, 34.2%), and nausea (*n* = 31, 11.2%) as possible side effects following ECT.

**Conclusion:**

In this study, ECT was found to decrease depressive and suicidal symptoms in adolescents, and the side effect was acceptable. ECT showed better outcome for MDD with SA compared to MDD with SI.

## Graphical Abstract

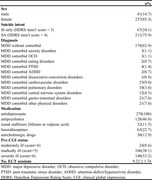

## 1 INTRODUCTION

Major depressive disorder (MDD) is a serious psychiatric disorder with high prevalence, high recurrence rate, and high death rate (García‐Peña et al., 2013; Hinrichsen & Hernandez, 1993), which is common in adolescents, the lifetime prevalence of MDD among 13‐ to 18‐year‐olds is 11.0% in the United States, with a 12‐month prevalence of 7.5% (Avenevoli et al., 2015). Suicide is defined as death caused by self‐directed injurious behavior with an intent to die as a result of the behavior and as a serious ill effect of MDD. Suicide in adolescents has become an increasingly potent public health and social issue all over the world. Suicide attempt (SA) is defined as a nonfatal, self‐directed, potentially injurious behavior with an intent to die as a result of the behavior even if the behavior does not result in injury, and suicidal ideation (SI) is defined as thinking about, considering, or planning suicide. Both of them belong to common suicidal symptoms. Among a survey of thousands of teenagers by JAMA Psychiatry in 2013 found that 1/8 had SI (Nock et al., 2013). In America, suicide leads the 3rd cause of death among teenagers, in which 4% of teenagers aged 10–18 have SA (Centers for Disease Control & Prevention, 2011). Therefore, it is of utmost significance to evaluate the suicidal symptoms in MDD adolescents.

Electroconvulsive therapy (ECT) is considered effective for mood disorders, especially with suicidal symptoms and catatonic symptoms (Benson et al., 2019; Puffer et al., 2016), and the side effect is also acceptable. However, the usage of ECT in adolescents is limited and has always been carefully evaluated. Previous studies showed adolescents were less likely to receive ECT among all age groups (Chanpattana et al., 2010). In some Asian studies, it was found that for patients younger than 18 years old, only about 6% would receive ECT (Rey & Walter, 1997). In Western studies, only 0.2%−1.5% of patients under 18 years old received ECT (Saatcioglu & Tomruk, 2008; Thompson & Blaine, 1987). One study from China found for adolescents, only 3.2% (406/12,608) of those would receive ECT (Zhang et al., 2016); the reason might be the misunderstanding of ECT or people worried about the side effect of ECT. In 2004, the AACAP developed practice parameters to assist clinicians considering ECT in treating adolescent patients. In these guidelines, criteria were outlined for selecting patients who might benefit from ECT treatment. Patients must have (1) a diagnosis of severe major depressive disorder, mania, schizoaffective disorder, schizophrenia, catatonia, or neuroleptic malignant syndrome, (2) symptom severity that is persistently disabling or life threatening, and (3) failure of at least two adequate prior treatment regimens when feasible (Ghaziuddin et al., 2004).

Although there have been no controlled studies of ECT in adolescents with depression and suicidal symptoms, some systematic reviews (Lima et al., 2013; Rey & Walter, 1997) and case series from ECT centers (Maoz et al., 2018; Puffer et al., 2016) suggested comparable efficacy in adolescent and young adult populations with the general adult population. For MDD adolescents with suicidal symptoms, previous studies showed worse outcome compared with MDD without suicidal symptoms (Grover et al., 2013; Puffer et al., 2016; Zhang et al., 2012), but we don't know the response rate of different suicidal symptoms, such as SI and SA, and if they showed different clinical outcome based on ECT. Sienaert et al. (2022) found the response rate of ECT for MDD adults with SI was 53.7%, but in MDD adults without SI, the response rate was 68.5%, and they did not discuss the response rate of SA. To our best knowledge, there are no randomized controlled trials to determine the efficacy of ECT in adolescents with depression and suicidal symptoms, so we try to find out the efficacy and safety in a retrospective series of adolescents hospitalized with different suicidal symptoms.

## 2 METHODS

### 2.1 Participants

We assessed 362 adolescents with depression and suicidal symptoms who took ECT during the years 2015 to 2021, The inclusion criteria were as follows: (1) age of 12 to 17 years; (2) a current diagnosis of major depressive episode diagnosed using the Diagnostic and Statistical Manual of Mental Disorders, Fourth Edition, Text Revision criteria and the 17‐item Hamilton Depression Rating Scale; (3) the third item of the HDRS ≥3; and (4) a scheduled ECT treatment of 6–12 sessions (Tran et al., 2017). Finally, 278 could meet the inclusion of our study, the diagnose was determined by two senior psychiatrists by DSM‐IV and we collected the sociodemographic, clinical and treatment records from the information center at the First Affiliated Hospital of Chongqing Medical University.

All the participants had suicidal symptoms, with SI only or SA, whereby SI means active suicidal thoughts, threats, gestures, and never had experience of SA and SA means experienced attempted suicide. The study protocol was approved by the Human Research and Ethics Committee of the first affiliated hospital of Chongqing medical university (NO: 20214801).

### 2.2 Assessment

#### 2.2.1 Clinical global impression scale

The clinical global impression scale (CGI) is widely recommended in measuring the symptoms of mental illness. The clinical global impression scale severity (CGI‐S) subscale is always used to evaluate the severity of symptoms, which ranges from 0 to 7, with 0 means normal or not at all ill and 7 means extremely ill (Guy, 1976), in present study, CGI assessment was carried out by two trained psychiatrists before and after ECT.

#### 2.2.2 Hamilton depression rating scale

Hamilton depression rating scale (HDRS) is a 17‐item scale, commonly used to evaluate the severity of depressive symptoms (Hamilton, 1960). We utilized a 50% reduction in HDRS scores as an indicator of treatment response. The Chinese version of HDRS has been demonstrated reliable and valid (Zhao & Zheng, 1992). the item 3 of HDRS is implemented to evaluate the severity of suicide, there are 4 levels: 0 equals absent; 1 refers feels life is empty or is not worth living; 2 denotes recurrent thoughts or wishes of death of self; 3 means active suicidal thoughts, threats, gestures; and 4 to serious suicide attempt. Scores of 3 and 4 indicated expressed suicide intent (Guy, 1976).

#### 2.2.3 Electroconvulsive therapy

The patients who took modified bitemporal ECT, used the brief‐pulse, constant‐current apparatus Thymatron (TM) DGx (Somatics LLC, Lake Bluff, IL, USA) at the department of psychiatry at the First Affiliated Hospital of Chongqing Medical University. Before ECT, all the patients would take laboratory examination such as blood routine test, liver, kidney and thyroid tests, ECG, brain CT scan and were kept nil orally for 12 h. ECT was done under general anesthesia, 3 times in the first week, then 2–3 times in the following time. All the participants received 6–12 sessions of ECT were enrolled in order to control the possible impact because of wild variance of ECT sessions (Inagawa et al., 2022). The initial dosage was considered by age: energy percent = age × 0.7. The stimulation energy was adjusted based on the seizure time. The energy was increased by 5% in the subsequent treatment if the seizure time was <25 s (Kranaster et al., 2013). Anesthesia and muscle relaxation were performed with diprivan (1.5−2 mg/kg) and succinylcholine (0.5−1 mg/kg), respectively, and subjects were monitored until they were awoken after an ECT session and able to identify adverse effects such as subjective memory impairment, headache, or nausea/vomiting. Data on ECT‐related adverse effects (such as memory complaints) were questioned in a direct clinical interview by a psychiatrist who applied ECT and observation by psychiatric nurses during hospitalization. Informed consent for the use of ECT was written by adolescents and their legal caregivers.

#### 2.2.4 Statistical analysis

IBM SPSS Statistics, version 25.0, was used for the statistical analysis. The sociodemographic and clinical characteristics of participants were evaluated by the mean (*n*), percentage (%), and standard deviations (SD). Using Kolmogorov–Smirnov test, the distribution of data (Kolmogorov., 1933) was estimated. Groups pre/post‐ECT were used by paired *t*‐test or Wilcoxon test, independent groups were analyzed by the two‐sample *t*‐test or Mann–Whitney *U* test as appropriate. The level of significance was set at *p* < .05.

## 3 RESULTS

### 3.1 Sociodemographic profile

Two hundred and seventy‐eight subjects were finally included in our research, where 14.7% of the participants were males (*n* = 41), aged from 12 to 17 (mean ± SD = 15.41 ± 1.50 years). Diagnoses among the sample were MDD without comorbid (*n* = 174, 62.6%), Comorbid diagnoses were found in 104 patients (37.4%). Diagnoses among the sample were anxiety disorder (*n* = 3, 1.1%), obsessive‐compulsive disorder (OCD) (*n* = 3, 1.1%), posttraumatic stress disorder (PTSD) (*n* = 4, 1.5%), eating disorder (*n* = 2, 0.7%), attention‐deficit/hyperactivity disorder (ADHD) (*n* = 2, 0.8%), and dissociative‐conversion disorder (*n* = 1, 0.4%). For comorbid diagnoses with medical illness, cardiovascular disorders (*n* = 25, 9.0%), pulmonary disorders (*n* = 10, 3.6%), central nervous system (CNS) disorders (*n* = 12, 4.3%), gastrointestinal (GI) disorders (*n* = 21, 7.6%), other disorders (*n* = 21, 7.6%), the ECT sessions ranged from 6 to 12 (mean ± SD = 9.22 ± 1.74). All the patients received antidepressants, with sertraline (*n* = 182; 65.5%) the most prescribed. Many of the patients also took benzodiazepines, alprazolam commonly taken (*n* = 82; 29.5%). See details in **Table** **1**.

**TABLE 1:** Sociodemographic and clinical profile

	n%
**Sex**	
male	41 (14.7)
female	237 (85.3)
**Suicide intent**	
SI only (HDRS item 3 score = 3)	67 (24.1)
SA (HDRS item 3 score = 4)	211 (75.9)
**Diagnosis**	
MDD without comorbid	174 (62.9)
MDD comorbid anxiety disorders	3 (1.1)
MDD comorbid OCD	3 (1.1)
MDD comorbid eating disorders	2 (0.7)
MDD comorbid PTSD	4 (1.4)
MDD comorbid ADHD	2 (0.7)
MDD comorbid dissociative‐conversion disorders	1 (0.4)
MDD comorbid cardiovascular disorders	25 (9.0)
MDD comorbid pulmonary disorders	10 (3.6)
MDD comorbid central nervous system disorders	12 (4.3)
MDD comorbid gastrointestinal disorders	21 (7.6)
MDD comorbid other physical disorders	21 (7.6)
**Medication**	
Antidepressants	278 (100)
Antipsychotics	128 (46.0)
Mood stabilizers (lithium or valproic acid)	32 (11.5)
Benzodiazepines	63 (22.7)
Anticholinergic drugs	36 (12.9)
**Pre‐CGI status**	
Moderately ill (score = 4)	24 (8.6)
Markedly ill (score = 5)	106 (38.1)
Severely ill (score = 6)	148 (53.2)
**No. ECT sessions**	9.22 ± 1.74

MDD: major depressive disorder; OCD: obsessive compulsive disorder; PTSD: posttraumatic stress disorder; ADHD: attention‐deficit/hyperactivity disorder; HDRS: Hamilton Depression Rating Scale; CGI: clinical global impression.

### 3.2 Clinical profile

Based on the results of CGI‐S, HDRS, and HDRS item 3, ECT showed effectiveness by significant differences pre/post‐ECT (*p* < .001 in all measures) (see **Table** **2**).

**TABLE 2:** Pre/post‐ECT HRSD, HRDS item 3, and CGI‐S

	Pre‐ECT, mean ± SD	Post‐ECT, mean ± SD	p
HDRS	29.6 ± 2.89	13.12 ± 5.10	<.001*
HDRS item 3	3.76 ± 0.43	0.44 ± 0.82	<.001*
CGI‐S	5.45 ± 0.65	2.88 ± 0.70	<.001*

* Wilcoxon test.

### 3.3 Response to ECT and adverse effects

The response rate of all MDD participants was 52%, for MDD with SI was 49%, while in MDD with SA, the response rate was 54%. Subjective memory complaint was the most common side effect. All side effects, including headache, body pain, and delirium, were managed conservatively, most of the side effects remitted within 48 h, but 26 patients reported memory complaint for more than two months. See **Table** **3** for details about the response rate and side effects. Using the CGI‐S, we tried to find out difference in clinical outcome within the different subgroups, and no significant differences were found between sex and comorbid subgroup (Mann–Whitney *U* test, *p* >.05), but SA subgroup showed better outcome than the SI subgroup (Mann–Whitney *U* test, *Z* scores = −7.335, *p*< .001) (see **Table** **4**).

**TABLE 3:** Response rate to ECT and adverse events with ECT

Variable	No. patients	Response rate
MDD	278	52%
MDD with SI	67	49%
MDD with SA	211	54%
**Adverse effects of ECT**	**n (%)**	
Subjective memory complaint	189(68)	
Headache	150(54)	
Body pain	28(10.1)	
Delirium	95(34.2)	
Nausea/vomiting	31(11.2)	

**TABLE 4:** Comparison of changes in CGI‐S scores between different subgroups

	Mean ± SD	Mean ± SD	p
Sex	Male (n = 41)	Female (n = 237)	
	2.80 ± 0.92	2.52 ± 0.92	.101*
Comorbidity	Present (104)	Absent (174)	
	2.24 ± 0.89	2.59 ± 1.00	.082*
Suicide intent	SI (67)	SA (211)	
	1.88 ± 0.72	2.78 ± 0.88	<.001*

* Mann–Whitney U test.

## 4 DISCUSSION

Our study demonstrated that adolescents experienced significant decrease in depression and suicidal ideation scores pre‐to‐post of ECT. Although the proportion of ECT in adolescents is much lower than that in adults, previous studies in adolescents have shown similar results. Grover et al. (2013) had found in adolescents, the efficacy of ECT was 87.2% for depression. Consoli et al. (2010) found that in adolescent patients, the clinical response rate of depression with psychotic symptoms was 85%; without psychotic symptoms, it was 69%. Rey and Walter (1997) found the remission rate was 63% for depression. Karayağmurlu et al. (2020) found ECT was significantly effective (72%) in MDD adolescents. Our study focused on depression with suicidal symptoms in adolescents; the response rate of MDD was 52%, in MDD with SI, it was 49%, and in MDD with SA, it was 54%, which was relatively lower compared with previous studies. Suicide is considered a feature of worse effect of MDD patients taking ECT, previous studies confirmed this view. Rootes‐Murdy et al. (2019) found that in adolescents who are taking ECT, suicidal symptoms were associated with worse response. Sienaert et al. (2022) found the response rate of ECT for MDD adults with SI was 53.7%, but in MDD adults without SI, the response rate was 68.5%.

In our study, we evaluated the clinical outcome by CGI‐S scores and found no significant differences in subgroups like gender or comorbid, but SA subgroup showed better outcome than the SI subgroup. When we reviewed the existing research, we could not find any related study focusing on the superiority of ECT in SI versus SA in MDD adolescents evaluating by CGI‐S scores. Better response in SA subgroup in our study might be related to different neurobiological response between SA and SI. It is still unclear why and when suicidal thoughts progress to potentially lethal attempts; previous theories tried to understand the ideation to action framework. Joiner found perceived burdensomeness and thwarted belongingness were main factors leading to SI, and acquired capability for suicide would lead SI to SA (Joiner., 2005). O'Connor (2011) established integrated motivational‐volitional theory and showed defeat and entrapment would lead to SI, and capability, impulsivity, planning, access to means, imitation, and other volitional moderators were main factors causing progression from ideation to attempts. Klonsky and May (2015) found combination of pain and hopelessness, especially when pain exceeds connectedness were main factors causing SI, dispositional, acquired, and practical contributors to increased capacity for suicide were main factors causing progression from ideation to attempts. These were psychological theories differences between SI and SA. MRI studies were also aimed to understand the differences between SI and SA. A fMRI study focusing on the relationship between SI and SA in depressive adults showed decreased amplitude of low‐frequency fluctuation (ALFF) and degree centrality (DC) in a frontoparietal network comparing SA patients with only SI patients (Wagner et al., 2021); this could be a potential reason for the different responses to ECT treatment. The mechanism of ECT reducing suicide risk of adolescents was unclear. We previously found ALFF decreased significantly in the right precentral gyrus, and DC decreased in the left triangular part of the inferior frontal gyrus and increased in the left hippocampus in MDD adolescents with SI after ECT; moreover, the changed brain function correlates with clinical outcome (Li et al., 2021). Based on above‐mentioned studies, differences were found on both psychological theories and neuroimaging mechanism, and impulsivity may be a key factor leading to SA, especially in younger age groups (Szanto et al., 2002). Ghaziuddin et al. (2020) found ECT could reduce impulsivity in adolescents with severe mood disorders; this might be the reason that the response rate of SA group showed better.

We found groups between different sex and comorbid showed no significant differences of treatment outcome. Previous studies showed comorbid diagnoses such as personality disorders, especially cluster B personality traits (Ghaziuddin et al., 2020) or dissociative disorders, and suicidal ideation or behavior always meant lower response rate (Karayağmurlu et al., 2020). In our study, 15 of 104 comorbid diagnoses belong to Axis 1 disorders, none Axis 2 disorders, and 85.6% belongs to Axis 3 disorders. In our sample, due to careful assessment of ECT in the adolescents, the comorbidities in Axis 3 were relatively mild diseases, such as sinus arrhythmia, mild liver dysfunction, mild anemia, hyperuricemia, etc. This may be one of the reasons why the response rate was not significantly different from that of patients without comorbidities.

Consistent with previous studies, we found that ECT was a safe choice for adolescents, and side effects were acceptable; in our study, the main side effects were subjective memory complaint (*n* = 189, 68%), headache (*n* = 150, 54%), delirium (*n* = 95, 34%), nausea/vomiting (*n* = 31, 11%), and body pain (*n* = 28, 10%). Subjective memory complaint was the most common adverse effects of ECT, which are found frequently with bilateral pulse‐wave ECT, and this was also the main reason for restricted treatment. Generally, memory loss could recover in 2–3 months (Semkovska & McLoughlin, 2010), but some studies reported that some still had loss of memory even 3 years after ECT (Squire & Slater, 1983). One out of 200 patients might have severe memory loss caused by ECT (Fink, 1979). Individualizing stimulus, electrode placement, and parameter configuration were considered useful for decreasing memory loss (Petrides & Fink, 1996; Sackeim et al., 2009; Sienaert et al., 2009). In one previous study, the rate of headache was 54%; reports of headache occurred in 26% to 85% (Drew et al., 2005). Post‐ECT headache was always mild and self‐limiting. It peaked within 2 h and subsided within 24 h; for adolescents, severe headache was usual (Dinwiddie et al., 2010).

### 4.1 Limitation

This study has some limitations. First, being a retrospective chart review, there was no control group to compare the overall improvement of depressive symptoms in patients of the same age group not receiving ECT. Second, the parameters of ECT such as electric energy and the duration of the stimulus were not included in this study. Third, we measured suicide symptoms only by HDRS item 3, and not by some structured specific instruments such as Beck Scale for Suicide Ideation or Columbia‐Suicide Severity Rating Scale. Finally, the evaluation of side effect was not detailed, so further study is still needed.

## 5 CONCLUSION

The present study demonstrates that ECT can decrease depression and suicidal symptoms in MDD adolescents with SI or SA. We found SA subgroup showed better outcome than the SI subgroup. Although the side effects were common, no one developed serious or life‐threatening symptoms. The main strengths of this study are of a relatively large number of subjects and the discussion between SI and SA in MDD adolescents.

## ETHICAL APPROVAL AND CONSENT TO PARTICIPATE

This study was conducted under the Declaration of Helsinki. The study protocol was approved by the Human Research and Ethics Committee of the first affiliated hospital of Chongqing medical university. Written Informed consents were obtained from the study participants to start data collection.

## AUTHOR CONTRIBUTIONS

All authors listed in the manuscript contributed sufficiently to qualify for authorship: Xiaolu Chen and Yixiao Fu conceived the structure of the manuscript and wrote the manuscript. Qianhong Zou, Yiting Zhang, Yu Tian, and Yu Yan collected the data. Qibin Chen, Xiaoyue Qin, and Lei Zou analyzed the data. Bangshu Zhao monitored patients after ECT. Xiao Li designed the study and reviewed the manuscript. All authors have read and approved the final manuscript.

## CONFLICT OF INTEREST

The authors declare no conflicts of interest concerning this paper.

### PEER REVIEW

The peer review history for this article is available at https://publons.com/publon/10.1002/brb3.2795

## Data Availability

The data will be available upon request from the Dr Xiao Li. cd_value_code=text
